# Singular Coincidence

**Published:** 1844-09

**Authors:** 


*Singular Coincidence.*—After the translation of the work which we are now publishing in the library part of our Journal, had for the present number nearly gone through the press, we received a letter from Jesse W. Cook, M. D., an experienced and scientific dental practitioner of Cincinnati, Ohio, (enclosing a printed translation of the title page of the same work,) informing us that he had added notes and additional plates to it, and was about to have it published. Believing that the sale of the translation procured by Dr. Cook, would be so curtailed as not to remunerate him for the labor and expense he would incur in bringing it out, we, at once, on the receipt of his letter, wrote to him, stating that it was in the course of publication in the Journal. In reply, the doctor informed us, that inasmuch as we were publishing it, he should decline doing so. We regret now, that we had not selected some other work for our purpose, as we have no doubt that the value of this would have been greatly enhanced by the notes and additional plates which Dr. Cook proposed adding to it.—*Balt. Ed.*

